# Partial Prosthetic Mitral Valve Dehiscence: Transapical Percutaneous Closure

**DOI:** 10.5935/abc.20180028

**Published:** 2018-03

**Authors:** Catarina Ruivo, José Ribeiro, Alberto Rodrigues, Luís Vouga, Vasco Gama

**Affiliations:** 1 Centro Hospitalar de Leiria, Leiria - Portugal; 2 Centro Hospitalar Vila Nova de Gaia, Espinho - Portugal

**Keywords:** Endocarditis, Mitral Valve Insufficiency, Aortic Valve Insufficiency, Echocardiography,Transesophageal

An 80-year-old woman with a history of mitral and aortic prosthesis replacement with biological prostheses due to endocarditis presented worsening dyspnea. A transthoracic echocardiogram demonstrated a paravalvular regurgitation between the left ventricle and left atrial appendage. Given her high-risk surgery (EuroSCORE-II: 38%), a percutaneous approach was performed for definitive closure.

**Figure 1:**
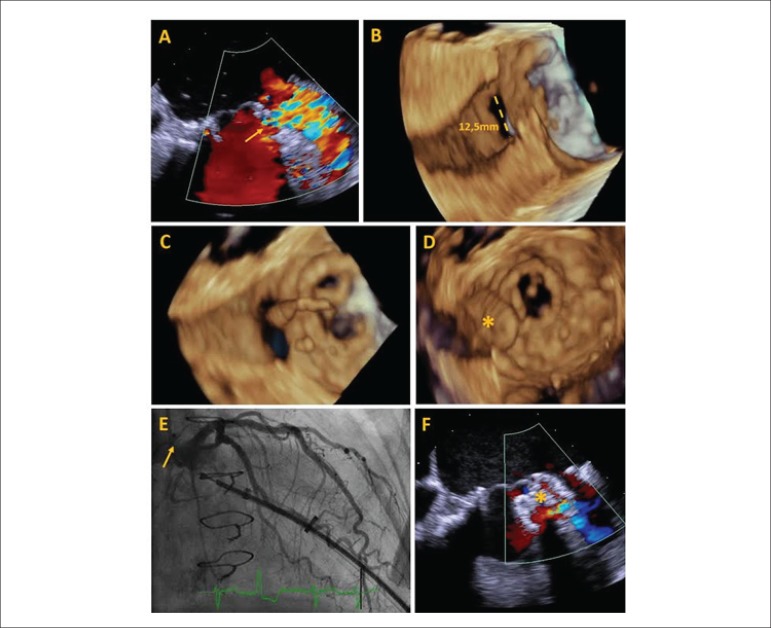
Panel A: 2D peri-processual transesophageal echocardiography (TEE) shows paravalvular regurgitation (yellow arrow) between left ventricle and left atrial appendage; Panel B: Defect 3D TEE with diameter measurement; Panel C: 3D TEE guiding the guidewire through the defect; Panel D: 3D TEE showing the device (asterisk) through the defect; Panel E: left coronary angiography without vascular involvement after occlusal implant (yellow arrow); Panel F: Light residual flux detected after device deployment (asterisk).

Transesophageal echocardiography (TEE) peri-procedure allowed the visualization of a partial dehiscence of the mitral prosthesis (Panel A, Figure 1). Through the 3D images, a tunneled defect with wall dissection measuring 12.5 mm of maximum diameter (Panel B, Figure 1) was observed. Using a transapical pathway and collecting three-dimensional (3D) images in real time, a 12 mm Amplatzer septal prosthesis was positioned, occluding the entire defect. The TEE 3D image demonstrated savings of adjacent structures and absence of pericardial effusion during closure. Coronary angiography demonstrated no arterial compromise. A slight residual flow was detected after device implantation (C-F Panels Figure 1).

Paravalvular regurgitation may result from suture dehiscence of the mitral prosthesis. Symptoms of heart failure are an indication for closure. A transapical approach allows direct access to the defect, providing good technical support. The TEE 3D image is essential for guiding the guidewire through the defect, confirming the correct position of the device and relating it to critical structures. The anatomy of the defect and the surrounding structures make this case a challenge, on both imaging acquisition and percutaneous technique

